# Human cerebrospinal fluid monoclonal CASPR2 autoantibodies induce changes in electrophysiology, functional MRI, and behavior in rodent models

**DOI:** 10.1016/j.bbi.2024.08.027

**Published:** 2024-08-13

**Authors:** Scott van Hoof, Jakob Kreye, César Cordero-Gómez, Julius Hoffmann, S. Momsen Reincke, Elisa Sánchez-Sendin, Sophie L. Duong, Manoj Upadhya, Divya Dhangar, Paulina Michór, Gavin L. Woodhall, Maraike Küpper, Andreas Oder, Joseph Kuchling, Stefan Paul Koch, Susanne Mueller, Philipp Boehm-Sturm, Jens Peter von Kries, Carsten Finke, Timo Kirschstein, Sukhvir K. Wright, Harald Prüss

**Affiliations:** a German Center for Neurodegenerative Diseases (DZNE) Berlin, 10117 Berlin, Germany; b Department of Neurology and Experimental Neurology, Charité-Universitätsmedizin Berlin, Corporate Member of Freie Universität Berlin, Humboldt-Universität Berlin, 10117 Berlin, Germany; c Helmholtz Innovation Lab BaoBab (Brain Antibody-omics and B-cell Lab), Berlin, Germany; d Department of Pediatric Neurology, Charité–Universitätsmedizin Berlin, corporate member of Freie Universität Berlin and Humboldt-Universität zu Berlin, Berlin, Germany; e Berlin Institute of Health at Charité – Universitätsmedizin Berlin, Charitéplatz 1, 10117 Berlin, Germany; f Institute of Health and Neurodevelopment, College of Health and Life Sciences, Aston University, Birmingham, UK; g Department of Paediatric Neurology, The Birmingham Women’s and Children’s Hospital National Health Service Foundation Trust, Birmingham, UK; h Oscar Langendorff Institute of Physiology, University of Rostock, Germany, Center of Transdisciplinary Neurosciences Rostock (CTNR), Germany; i Screening Unit, Leibniz Forschungsinstitut für Molekulare Pharmakologie, 13125 Berlin, Germany; j Neurocure Cluster of Excellence, NeuroCure Clinical Research Center, Charité – Universitätsmedizin Berlin, corporate member of Freie Universität Berlin and Humboldt-Universität zu Berlin, Germany; k Center for Stroke Research Charité Universitätsmedizin Berlin, corporate member of Freie Universität Berlin and Humboldt-Universität zu Berlin, Germany; l NeuroCure Cluster of Excellence and Charité Core Facility 7T Experimental MRIs, Charité – Universitätsmedizin Berlin, Germany; m Charité 3R, Replace, Reduce, Refine, Charité – Universitätsmedizin Berlin, Germany

**Keywords:** CASPR2, Autoimmune encephalitis, Autoantibodies, Human monoclonal antibody, Mouse MRI

## Abstract

Anti-contactin associated protein receptor 2 (CASPR2) encephalitis is a severe autoimmune encephalitis with a variable clinical phenotype including behavioral abnormalities, cognitive decline, epileptic seizures, peripheral nerve hyperexcitability and neuropathic pain. The detailed mechanisms of how CASPR2 autoantibodies lead to synaptic dysfunction and clinical symptoms are largely unknown. Aiming for analyses from the molecular to the clinical level, we isolated antibody-secreting cells from the cerebrospinal fluid of two patients with CASPR2 encephalitis. From these we cloned four anti-CASPR2 human monoclonal autoantibodies (mAbs) with strong binding to brain and peripheral nerves. All were highly hypermutated and mainly of the IgG4 subclass. Muta-genesis studies determined selective binding to the discoidin domain of CASPR2. Surface plasmon resonance revealed affinities with dissociation constants K_D_ in the pico- to nanomolar range. CASPR2 mAbs interrupted the interaction of CASPR2 with its binding partner contactin 2 *in vitro* and were internalized after binding to CASPR2-expressing cells. Electrophysiological recordings of rat hippocampal slices after stereotactic injection of CASPR2 mAbs showed characteristic afterpotentials following electrical stimulation. *In vivo* experiments with intracerebroventricular administration of human CASPR2 mAbs into mice and rats showed EEG-recorded brain hyperexcitability but no spontaneous recurrent seizures. Behavioral assessment of infused mice showed a subtle clinical phenotype, mainly affecting sociability. Mouse brain MRI exhibited markedly reduced resting-state functional connectivity without short-term structural changes. Together, the experimental data support the direct pathogenicity of CASPR2 autoantibodies. The minimally invasive EEG and MRI techniques applied here may serve as novel objective, quantifiable tools for improved animal models, in particular for subtle neuro-psychiatric phenotypes or repeated measurements.

## 1 Introduction

Contactin-associated protein receptor 2 (CASPR2) is essential for the physiological function of nerve fibers where it contributes to the positioning of voltage-gated potassium channel (VGKC) complexes. Through the interaction with contactin 2 on myelinating cells, it localizes the VGKC complex at the juxtaparanodal regions of an axon, allowing proper repolarization following an action potential (Saint-Martin et al., 2018). Genetic absence of CASPR2 accordingly leads to a severe phenotype in humans, including intellectual disability and epilepsy (Rodenas-Cuadrado et al., 2016).

Similar to other proteins of the synapse, CASPR2 can become the target of antibody-mediated autoimmunity (Irani et al., 2010; Prüss, 2021). Anti-CASPR2 autoantibody encephalitis (CASPR2 encephalitis) occurs typically in older men and is characterized by a wide range of syndromes, from limbic encephalitis with cognitive decline and seizures, cerebellar ataxia to Morvan syndrome, which includes peripheral nerve hyperexcitability, severe insomnia, and encephalopathy (Van Sonderen et al., 2016). The progression of this disease, unlike other forms of autoimmune encephalitis, is often slow with patients at first presenting with unspecific neurological or psychiatric symptoms (Benoit et al., 2023). Most patients respond to immunotherapy, supporting a direct pathogenic role for the autoantibodies (Van Sonderen et al., 2016). Previous *in vitro* work using serum or cerebrospinal fluid (CSF)-derived IgG fractions suggested that polyclonal CASPR2 autoantibodies can interrupt the association between contactin 2 and CASPR2 (Joubert et al., 2022; Patterson et al., 2018) and can disrupt α-amino-3-hydroxy-5-methylisoxazole-4-propionic acid (AMPA) receptor functioning at the synaptic level (Fernandes et al., 2019). Infusion of patient-derived IgG into the brain of mice yielded reversible alterations of memory and social behavior (Giannoccaro et al., 2019; Joubert et al., 2022). The majority of studies focused on the effects of polyclonal autoantibodies. Only one study utilized a blood-derived monoclonal CASPR2 autoantibody from a person without CASPR2 encephalitis and demonstrated that, when embryos were exposed in utero, it caused abnormal cortical and neuronal development and impairments in social behavior (Brimberg et al., 2016).

Therefore, the present study aimed at generating and characterizing a repertoire of CSF-derived human monoclonal antibodies (mAbs) from B cells of patients with CASPR2 encephalitis. We hypothesized that CASPR2 mAbs exhibit unique binding characteristics, leading to pathogenic effects detected both *in vitro* and *in vivo*. Using a multi-step approach, CASPR2 mAbs were recombinantly produced and analyzed for their antigenic epitopes, IgG subclasses, and binding characteristics. Functional effects were assessed *in vitro* and *in vivo*, including electrophysiology, magnetic resonance imaging and behavioral analyses.

## 2 Materials and methods

### 2.1 Patient sample collection and handling

The patients (n = 2, 60 and 66 years of age) gave written informed consent for sample processing. The analyses were approved by the Charité University Hospital Institutional Review Board (#EA1/258/18). Five ml of CSF was collected during the acute encephalitis phase, and immediately processed for cell cryopreservation. For this, centrifugation at 400x g was performed for 10 min, the supernatant was taken and stored at −80 °C. The remaining cell pellet was resuspended in 500 μl of 10 % dimethyl sulfoxide, 45 % fetal calf serum and 45 % RPMI medium, and then frozen at −80 °C.

### 2.2 Single cell sorting

Fluorescence activated cell sorting was performed as described in (Kreye et al., 2021). In brief, lymphocytes (CD3^-^, CD14^-^, CD16^-^, DAPI^-^) were sorted into 96-well PCR plates containing hypotonic lysis solution and categorized as antibody secreting cells (ASCs, CD138^+^), memory B cells (MBC, CD20^+^ CD27^+^) and non-memory B cells (NMBCs, CD20^+^ CD27^-^), respectively. The following antibodies were used for staining: anti–CD3-FITC (1:25; Miltenyi Biotec; #130–098-162), anti–CD14-FITC (1:25; Miltenyi Biotec; #130–098-063), anti–CD16-FITC (1:25; Miltenyi Biotec; #130–098-099), anti–CD20-PerCP-Vio700 (1:50; Miltenyi Biotec; #130–100-435), anti–CD27-APC-Vio770 (1:12.5; Miltenyi Biotec; #130–098-605), and anti–CD138-PE (1:50; Miltenyi; #130–098-122).

### 2.3 Recombinant human mAbs generation

Recombinant human monoclonal antibodies were generated from sorted cells as described previously (Kornau et al., 2020; Kreye et al., 2021, 2016). In short, cDNA was produced from individual cells, and the genes encoding the variable domains of immunoglobulin (Ig) heavy and light chains were amplified and sequenced. They were cloned into expression vectors containing the constant domains of the respective chain and analyzed for genetic characteristics and Ig subclasses using BASE software (Reincke et al., 2020). A pair of Ig expression vectors was transiently transfected using HEK293T cells. Supernatant containing the Ig was harvested, and CASPR2 antibodies were purified for further characterization as described before (Kreye et al., 2016).

### 2.4 Reactivity screening

For CASPR2 reactivity screening in a cell-based assay, HEK293T cells were cultured on poly-L-lysine-coated coverslips and transiently transfected with human *CNTNAP2* containing a cmyc-DDK tag (Origene, CAT#:RC210836). After 2 days, cells were fixed with 4 % para-formaldehyde for 10 min and washed 3 x 5 min with PBS. Cells were incubated with blocking solution (in PBS: 5 % NGS, 2 % BSA, 0.05 % NaN_3_, 0.1 % Triton) for 1 h, followed by undiluted supernatants over-night at 4 °C. After washing, Alexa488-labeled goat anti-human IgG (1:1000; Dianova CAT#:109–545-003) was added for 2 h, cells were washed, and mounted on Superfrost™ slides using Immu-Mount™ medium (Epredia). CASPR2 reactivity was confirmed with double-staining of the c-myc-DDK tag (mouse anti-myc-DDK, 1:100; OriGene CAT#: TA150121, and Alexa594-labeled goat anti-mouse IgG secondary anti-body, 1:1000; Jackson ImmunoResearch CAT#:115–585-003). Images were obtained on a Leica SPE using confocal imaging.

Neuronal reactivity of all recombinant mAbs was further screened on mouse brain tissue. Frozen mouse brains were cut in 20 μm sections and mounted on glass slides. Cryo-sections were rinsed with PBS and fixed with 4 % paraformaldehyde for 10 min. After washing with PBS, sections were incubated in blocking solution (in PBS: 5 % NGS, 2 % BSA, 0.05 % NaN_3_) for 1 h at room temperature (RT). The supernatants were then stained as described above.

### 2.5 Sciatic nerve staining

Sciatic nerves were harvested from sacrificed 10–12 days old C57Bl/6J mice, fixed in 500 μl of 4 % PFA for 20 min on ice, and washed three times with ice-cold PBS. Using a stereo microscope (Leica EZ4W) and tweezers, the nerves were teased on Superfrost™ Plus slides (Epredia). Teased fibers were dried overnight at 4 °C, and then stored at − 20 °C. For staining, the fibers were postfixed in methanol for 3 min at − 20 °C and washed with PBS. Staining was performed as described above using the purified recombinant CASPR2 mAbs, double-labeled with rabbit anti-mouse K_v_1.2 (1:200; Neuromab CAT#: 75–008). Secondary anti-bodies were Alexa488 goat anti-human (1:1000; Dianova) and Alexa594 goat anti-rabbit IgG (1:1000; Jackson ImmunoResearch).

### 2.6 Epitope mapping

To determine CASPR2 binding epitopes of patient-derived mAbs, several constructs were generated using human full-length CASPR2 cDNA as a template. Eight deletion constructs were generated using the Q5 Site-Directed mutagenesis kit (NEB), each construct has one domain deleted. For this, primer pairs flanking individual CASPR2 domains were designed. Individual domains were deleted according to the manufactureŕs protocol and confirmed through Sanger sequencing. In addition, a construct was generated which included the discoidin domain only. Human mAbs reactivity against the CASPR2 mutants was determined using CBA.

### 2.7 Binding strength determination

For evaluation of mAb binding strength, protein high-binding 96-wells plates were coated with 100 ng/well of recombinant human CASPR2 protein (R&D) in binding buffer (20 mM Tris, pH 8.0, 100 mM NaCl, 5 mM CaCl_2_) at 4 °C overnight. Plates were washed 3x with binding buffer and incubated with blocking buffer (1 % BSA in binding buffer) for 1 h at RT. After washing, CASPR2 mAbs were added in a 10-fold dilution series (0.05–50 μg/ml in binding buffer containing 0.1 % Tween) and incubated for 1 h at RT After washing, horseradish peroxidase (HRP) labelled goat anti-human IgG (1:25000) was added for 1 h at RT. The reaction was started with Ultra-TMB (Thermofisher) and stopped after 5 min with 2 M H_2_SO_4_. The absorbance was measured at 450 nm using a TECAN reader. Three independent repetition experiments were performed, and all samples measured in duplicates.

### 2.8 Surface plasmon resonance

Autoantibodies affinity to CASPR2 was evaluated using surface plasmon resonance on a Biacore T200 instrument at 25 °C. Purified mAbs were reversibly immobilized via the anti-human IgG capture surface. The CASPR2 protein (R&D CAT#:8207-CR) was injected at different concentrations in a buffer consisting of 10 mM HEPES pH 7.4, 150 mM NaCl, 3 mM EDTA, 0.05 % Tween-20 and 0.1 mg/ml BSA. K_a_, K_d_ and K_D_-values were determined using a monovalent analyte model.

### 2.9 Antibody effect on CASPR2/contactin 2 interaction in vitro

To measure the effect of CASPR2 mAbs on the CASPR2/contactin 2 interaction, a modified protocol was used (Patterson et al., 2018). Plates were incubated with CASPR2 protein as described above, washed and incubated for 1 h at RT with 100 ng of contactin 2 (Sino Biological, Beijing, China). CASPR2 mAbs or isotype control were added in dilution series for 1 h. After washing, mouse anti-human contactin 2 antibody (1:100; R&D) was added for 1 h at RT, followed by HRP-labelled antimouse IgG for 1 h. After washing, reaction was developed with Ultra-TMB for 20 min, stopped with 2 M H_2_SO_4_, and extinction coefficient measured at 450 nm. Background signal of isotype controls was subtracted, and the percentage of inhibition was calculated relative to a control condition without antibody.

### 2.10 Internalization of CASPR2 through antibody binding

To determine the potential of CASPR2 mAbs to cause CASPR2 internalization, we used the pH-sensitive dye pHrodo for detection of transport into the acidic endosomal compartment. The antibodies were conjugated with pHrodo Red Succinimidylester (Invitrogen, P36600) according to manufacturer instructions, and applied for 4 h or 24 h at 37 °C on HEK293T cells that were either transfected with human CASPR2, or sham transfected. After incubation, cells were washed with 1x PBS and fixed with PFA (4 %), before mounting with Prolong Gold Antifade mounting medium (Invitrogen, P36934). Images were taken in both bright field and 561 nm absorption fluorescence microscopy using a Nikon CSU-W1 SoRa spinning disk confocal microscope. After acquisition, images were processed using Fiji is just ImageJ (FIJI).

### 2.11 Animals

All animal experiments were performed according to ARRIVE guidelines, UK Home office guidelines and German guidelines for care and humane use of animals. 10–12-weeks old male C57BL/6 mice, 21-day old male Wistar rats and 6–9-week old female Wistar rats were used. All animals were housed in a humidity and temperature-controlled environment with a 12 h/12 h dark/light cycle. The animals were provided with food and water ad libitum. Studies involving intrathecal antibody administration were approved by the Landesamt für Gesundheit und Soziales (LaGeSo) in Berlin, Germany (approval number G0078/19), and the Landesamt für Landwirtschaft, Leb-ensmittelsicherheit und Fischerei Mecklenburg-Vorpommern (LALLF) in Rostock, Germany (approval number M−V/TSD/7221.3–1.1–007/16). For the rat studies, the *in vivo* EEG recordings and intra-cerebroventricular infusion procedures were carried out under the authority of a UK Home Office approved project license (P9FA3BDD1), and local ethical approval for the study was granted by the Aston Bioethics Committee, University of Aston, Birmingham, UK.

### 2.12 Ex vivo hippocampal slice recordings

Prior to *ex vivo* hippocampal slice recordings, rats were stereotactically injected *in vivo* (Blome et al., 2018). To anaesthetize, 6–9 week-old female Wistar rats were administered S-ketamine (100 mg/kg i.p.) and xylazine (15 mg/kg i.p.), and mounted on a stereotactic frame (Narishige, Tokyo, Japan) to inject either CASPR2 mAbs in PBS (n = 4) or vehicle (n = 4). The injection was performed in 10 steps of 0.5 μl every 2 min (i.e. 5 μl for each side) using a Hamilton syringe (75 N; Hamilton AG, Bonaduz, Switzerland). The coordinates for the stereotactic injection were: 5.2 mm posterior, ±4.3 mm lateral, 4.8 mm deep (relative to bregma) (Blome et al., 2018). After surgery, rats received metamizole (100–150 mg/kg) to control postoperative pain and were allowed to recover in an enhanced O_2_ atmosphere (4–5 l/minute in an 8 l glass vessel).

*Ex vivo* electrophysiology was carried out as described previously (Küpper et al., 2023). In short, hippocampal slices were prepared 2–7 days after stereotactic injection of antibody or control, after which extracellular recordings were performed, measuring field excitatory postsynaptic potentials (fEPSP) and their potential epileptiform after-potentials after stimulation (25-200μA). At the beginning of the experiment, input–output relationships were recorded, and a paired pulse paradigm was used, which after short-term plasticity induction, was again obtained. Analogue data were amplified and filtered, digitized and then analyzed as described in previously (Küpper et al., 2023).

### 2.13 Rat in vivo electrophysiology

To determine epileptogenic activity of CASPR2 mAbs *in vivo*, rats were infused with CASPR2 mAbs as described previously (Kreye et al., 2021; Wright et al., 2021). In short, juvenile Wistar rats were infused intracerebroventricularly with either 300 μg mAb HK187-194 (n = 6) or control antibody (n = 5) over a 7-day period with simultaneous CA3 24-hour wireless depth EEG recording over a 21-day period after which the animals were sacrificed and brains were harvested for histological analysis. Detailed collection and analysis of the EEG data is described previously (Kreye et al., 2021; Wright et al., 2021). Statistical significance was determined using Mann-Whitney *U* test.

### 2.14 Mouse intrathecal osmotic pump infusion

Mice were randomized for the treatment groups by an independent investigator. They received 200 μg (2 μg/μl IgG) of mGO53 (control) (n = 14) or HK187-194 (CASPR2 mAb) (n = 14) into the right lateral ventricle using osmotic minipumps over 14 days, the maximum infusion time with the model used (model 1002, Alzet, Cupertino, CA). For pump implantation, mice were placed in a stereotaxic frame and a cannula was inserted into the right ventricle (coordinates: 0.2 mm posterior and ± 1.00 mm lateral from bregma, depth 2.2 mm). The cannula was connected to a pump, which was subcutaneously implanted in the inter-scapular space. After surgery, mice were monitored daily to assess symptoms and weight variations. Mice were sacrificed after 14 days and brains were post fixed (4 % PFA) overnight. For quantification of mAb binding, mean fluorescence intensity (MFI) of Alexa488 goat anti-human (1:500 Dianova) staining was calculated from 20 randomly selected areas of three sections per animal (n = 6/group) using FIJI is just ImageJ (FIJI).

### 2.15 Behavioral analysis

Behavioral tests were conducted 10–14 days after pump implantation. Selection of tests was based on an exploratory approach to cover anxiety, motor function, memory and social behavior in this short time period. All tasks were performed and assessed by trained scientists under blinded conditions. Prior to testing, all animals were given > 30 min for habituation. All variables were tested for significance using unpaired *t*-test, except for the three chamber test, which used paired t-tests.

#### 2.15.1 Open field test

For assessment of general locomotor activity levels, anxiety, and willingness to explore unfamiliar environments, one animal at a time was placed in an empty arena (50 x 50 cm) and left to explore it for 10 min. During exploration, time spent in the center, speed of movement and the number of visits in the center were measured.

#### 2.15.2 Rotarod

Rotarod apparatus (Panlab, LE8205) was used for evaluation of motor coordination and balance. Mice were tested in 3 trials with an inter-trial interval (ITI) of 15 min, speed of the rotating rod accelerated from 4 to 40 rpm within 300 sec. Elapsed time until the animals fall off the rod is measured.

#### 2.15.3 Y-maze

The spontaneous alternation test measures the willingness to explore new environments. Testing was conducted in a Y-shaped maze with three arms at a 120° angle from each other. Mice were placed in one arm and allowed to explore the maze for 5 min. The starting arm was alternated between animals.

#### 2.15.4 Three-chamber test

The three-chamber test consists of 3 phases and assesses social interaction and the preference for social novelty. During phase 1, animals habituated and freely explored the tripartite chamber for 10 min. In phase 2, animals were presented an unknown mouse placed in a grid enclosure at one of the lateral chambers, allowing close interaction. The natural interest for a novel subject was evaluated based on the time in close proximity to the stranger mouse, the number of visits to the novel animal, and the latency to make contact. During phase 3, a second unknown mouse was placed at the other side of the chamber. Again, time spent with the unfamiliar mouse, latency and number of contacts were recorded.

### 2.16 MRI acquisition

Anesthesia was achieved using 1.5 %-2% isoflurane in a 70:30 nitrous oxide:oxygen mixture. Body temperature, and respiration rate were monitored throughout the experiment with MRI compatible equipment (Small Animal Instruments Inc., Stony Brook, NY, USA). Anatomical images were acquired on a 7 T MR scanner (Bruker Biospec 70/20 USR, Ettlingen, Germany) and a transmit/receive mouse ^1^H cryoprobe (Bruker) using a 2D turbo spin echo pulse sequence (2D RARE, repetition time (TR) = 4250 ms, effective echo time (TE) = 33 ms, echo time spacing (ΔTE) = 11 ms, RARE factor 8, 2 averages, 40 contiguous 0.4 mm thick coronal slices, field of view (FOV) = 19.2x19.2 mm^2^, image matrix (MTX) = 192x192, total acquisition time (TA) = 3:26 min). Before start of the resting statefunctional MRI (rs-fMRI) scan, isoflurane levels were reduced to 1.2 (+/- 0.3) % until animals breathed regularly at higher rate of 160 (+ /-25)/min. This process took 5–7 min.2D echo-planar imaging (EPI) rsMRI images were acquired (TR=1000 ms, TE=13 ms, flip angle (FA) = 50°, 300 repetitions, 16 contiguous 0.75 mm thick coronal slices, FOV=19.2x12.0 mm^2^, MTX=128 x 80, TA=5:00 min) Thereafter, isoflurane was increased back to 1.5 %-2% and diffusion MRI (dMRI) was performed using a spinecho EPI sequence (4 segments, TR=3500 ms, TE=30 ms, 60 diffusion directions with b =1000 s/mm^2^, 5b =0 images, gradient duration/ separation =2.7 ms/8.6 ms, geometry matching the anatomical MRI, MTX=160x160, TA=15:10 min) Experimenters were blinded to the condition of the animals.

### 2.17 Anatomical MRI analysis

The anatomical MR image was segmented into gray matter (GM), white matter and cerebrospinal fluid probability maps and registered to the Allen brain atlas using ANTx2 (https://github.com/ChariteExpMri/antx2) as described previously (Hikishima et al., 2017; Klein et al., 2010; Koch et al., 2019; Lein et al., 2007). Voxel-based morphometry (VBM) group comparisons were performed on GM maps in SPM8 (https://www.fil.ion.ucl.ac.uk/spm/software/spm8/) using cluster-based analysis to correct for multiple comparisons. The reverse registration was used to segment brain areas on the original MR images. Volumes of individual brain areas of the Allen atlas were expressed in percent of whole brain volume and compared between groups.

### 2.18 rs-fMRI analysis

Processing of rs-fMRI data was modified according to an established rs-fMRI processing pipeline and analogous to a previously published protocol (Hall et al., 2022). Two image sets had to be excluded from processing due to insufficient data quality (one animal from CASPR2-ab group and one animal from control group).

Resting state networks common to all mice were identified for each cohort, separately, by use of temporal-concatenation independent component analysis (ICA) with automatic component estimation as implemented in FSL MELODIC. To facilitate classification, resulting components were displayed as spatial color-coded z-maps onto the Allen Mouse Brain atlas (AMBA; mouse.brain-map.org/static/atlas) after co-registeration with FSL FLIRT. Group comparisons were carried out using dual regression and nonparametric permutation testing (1,000 permutations) with threshold-free cluster enhancement (TFCE) as implemented in FSL *randomise* (p < 0.05, familywise error [FWE]-corrected). Resultant maps from group analyses were co-registered to AMBA for visualization.

### 2.19 dMRI analysis

The b = 0 images were used to perform an affine registration of the anatomical image and overlaid atlas to the dMR images. The dMRI data was processed in mrtrix3 (https://www.mrtrix.org/), the detailed pipeline is openly available (https://github.com/ChariteExpMri/rodent DtiConnectomics/tree/main/mouse_singleShell_dtiConnectomics).

Briefly, images were preprocessed using denoising, corrections for bias field, Gibbs ringing and Eddy currents followed by constrained spherical deconvolution reconstruction. Using the registered atlas and probabilistic fiber tracking, connectivity matrices were generated. T-test of group differences of connectivity strength (CS) between brain areas were not significant after correcting multiple comparisons using false discovery rate. Therefore, an additional exploratory analysis was performed without post-hoc corrections and using a fixed threshold of p = 0.00001.

## 3 Results

### 3.1 Human monoclonal CASPR2 autoantibodies derived from CSF antibody-secreting cells and were mainly of the IgG4 subclass

To characterize the intrathecal antibody repertoire and to investigate CASPR2 autoantibody effects at the monoclonal level, CSF cells from two CASPR2 encephalitis patients were isolated via flow cytometry following our established protocols (Kornau et al., 2020; Kreye et al., 2020, 2016). Single cells were FACS-isolated based on the presence of B cell markers, i.e. CD27^+^CD38^+^ ASCs, CD20^+^CD27^+^ MBCs, and CD20^+^CD27^-^ NMBCs. The variable parts of Ig light and heavy chain genes from these cells were cloned into IgG1 vectors, and mAbs recombinantly expressed in HEK293T cells. Both patients had CASPR2 encephalitis with anti-CASPR2 autoantibodies in serum and CSF together with the compatible clinical picture (Supplementary Table 1). Patient 187 was negative for the DRB1*11:01 HLA haplotype, patient 219 was not tested. At the time of CSF puncture, patient 187 was untreated, while patient 219 had already received immunotherapy (prednisolone, rituximab, bortezomib, daratumumab). All patient-derived mAbs originated from ASC, indicating active autoantibody production at the time of the spinal tap (Table 1).

**Fig. 1:**
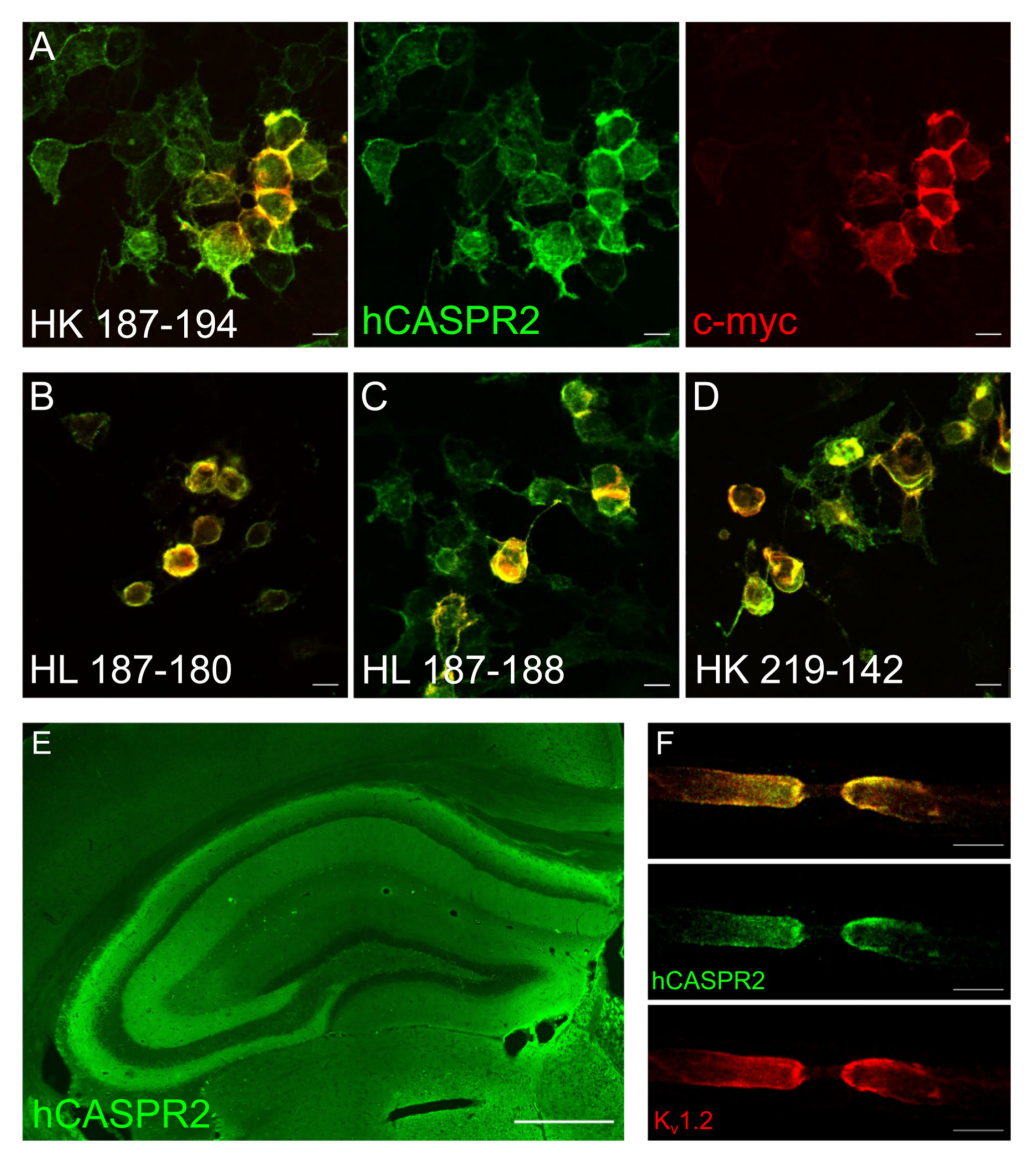
CASPR2 reactivity of isolated monoclonal antibodies. Cell based assay CASPR2 reactivity for HK 187–194 (A), HL 187–188 (B), HL 187–180 (C) and HK 219–142 (D), with human IgG shown in green, and c-myc-DDK tagged CASPR2 in red. Validation of CASRP2 reactivity shown in intrathecally infused mouse hippocampus (E) mouse teased sciatic nerves at 40x magnification for HK 187–194, with human IgG shown in green, and K_v_1.2 shown in red (F).

CSF-derived mAbs were then tested for reactivity against CASPR2 in a clinical routine cell-based assay (CBA) overexpressing human CASPR2 (Fig. 1A). Of eleven mAbs, five (HK187-113, HL187-180, HL187-188, HK187-194, and HK219-142) demonstrated strong binding in the CBA, co-localizing with the expression marker c-myc-DDK (Fig. 1A-D, Table 1). Two CASPR2 mAbs were identical (HK187-194 and HK187-113) indicating clonal B cell expansion (Table 1). CASPR2 reactivity was further confirmed by the characteristic anatomical staining pattern in the central and peripheral nervous system, i.e. on hippocampal brain sections (Fig. 1E) and the teased mouse sciatic nerve fibers, co-localizing with the potassium channel K_v_1.2 (Fig. 1F). Of the remaining six mAbs not binding CASPR2, four exhibited unique tissue staining patterns on unfixed mouse brain, in particular in the hippocampus and basal ganglia (Suppl. Fig. 1). The Ig subclass of two of these was IgM. The Ig subclass in three of the four CASPR2 mAbs was IgG4, and all CASPR2 mAbs showed high numbers of somatic hypermutations (SHM), indicating antigen-driven selection and affinity maturation (Table 1).Other mAbs Ig subclasses included an IgA that, while clonally expanded, was not reactive to CASPR2 or murine brain sections.

### 3.2 Autoantibodies bound to the discoidin domain of CASPR2 and disrupted its interaction with contactin 2

**Fig. 2:**
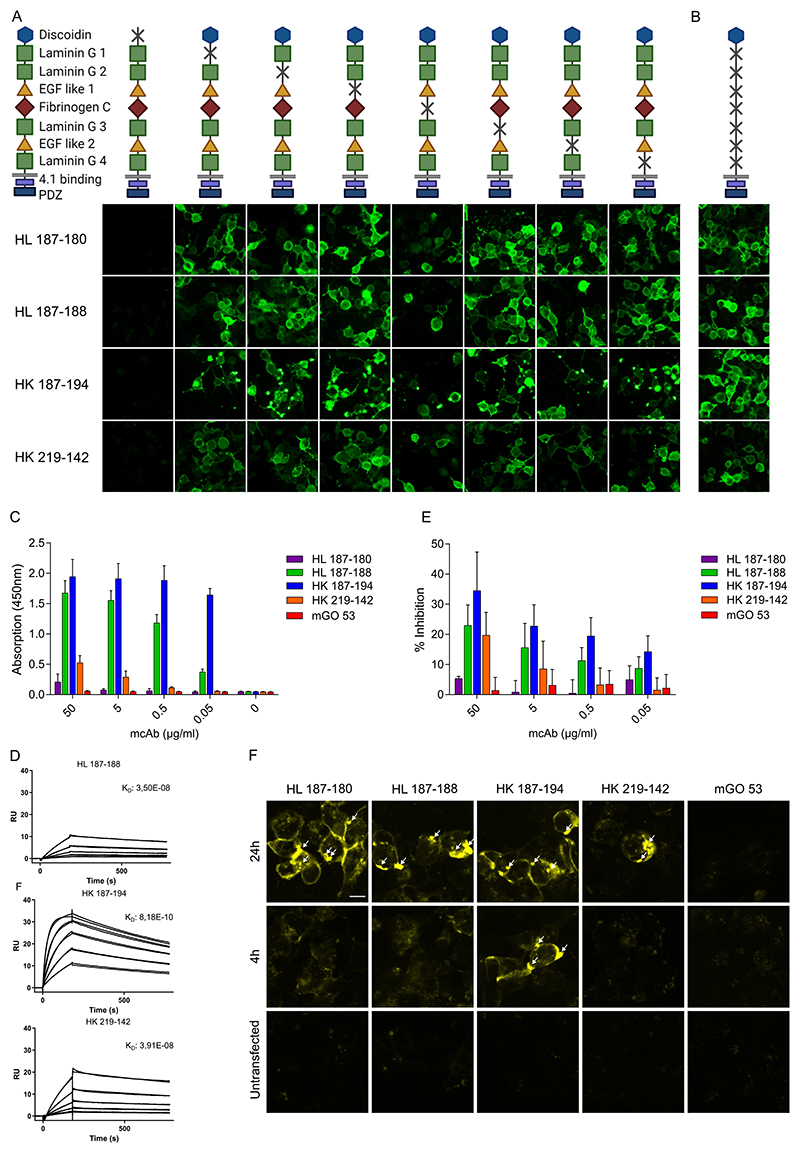
Binding characterization of CASPR2 reactive monoclonal antibodies. Schematic of CASPR2 protein, indicating the location of each domain and subsequent generated deletion constructs, shown below is reactivity to each corresponding construct through cell based assay, with green showing human IgG (A). Schematic of the discoidin isolation construct with the reactivity of each mAb in green shown below (B). Binding strength of the monoclonal antibodies as determined through a solid phase binding assay using human CASPR2 protein. Antibody concentration used ranged from 50 to 0.05 ug/ml. Bars indicate mean ± SEM (C). Affinity of three antibodies as determined by surface plasma resonance, using human CASPR2 protein, shown with kD values (D). Inhibition of contactin-2 binding to CASPR2 determined in solid phase binding assays (n = 3), here shown in percentage of contactin-2 inhibited compared to condition without antibody. Each antibody is shown in a range of concentrations, from 50 to 0.05 ug/ml. Bars indicate mean ± SEM (E). Internalization of pHrodo-coupled anti-CASPR2 mAbs after 4 h and 24 h incubation on CASPR2-transfected HEK293T cells. Untransfected cells were used as controls. Scale bar indicates 10 μm (F).

To investigate the antigenic epitopes of the human CASPR2 mAbs, we generated constructs using mutagenesis of the CASPR2 protein, each lacking a single domain (Fig. 2A). Constructs were expressed in HEK cells and stained with CASPR2 mAbs for epitope mapping, demonstrating that the presence of the discoidin domain was essential for antibody binding. In contrast, all other mutants had no effect on anti-body reactivity (Fig. 2A). CBA expressing the discoidin domain alone confirmed binding of all CASPR2 mAbs to this ‘main immunogenic region’ (Fig. 2B).

Next, we investigated the binding strength of the mAbs to human CASPR2 using a solid phase binding assay (Fig. 2C) and determined affinity through surface plasmon resonance (SPR) (Fig. 2D. Of the four CASPR2 mAbs, HK187-194 showed the strongest binding, still being clearly detectable at a concentration of 0.05 μg/ml (Fig. 2C). Accordingly, SPR analysis demonstrated a K_D_ value of 8.18E-10 for this anti-body, which corresponded to the highest number of SHM (Fig. 2D). In contrast, HL187-180 bound CASPR2 only at high concentrations of 50 μg/ml in the solid phase binding assay and was not detectable with SPR (data not shown). HL187-188 and HK219-142 showed intermediate binding strengths with K_D_ values of 3.5E-08 and 3.91E-08, respectively (Fig. 2C,D).

Given the necessity of CASPR2/contactin 2 interaction for the localization of the VGKC, we determined whether binding of the CASPR2 mAbs may interfere with this interaction. Indeed, depending on their affinity and concentration, the CASPR2 mAbs inhibited the binding of contactin 2. For the high affinity antibody HK187-194, inhibition ranged from 15-35 % (Fig. 2E, confirming a molecular mechanism of pathogenicity for the human CASPR2 mAbs.

Internalization of CASPR2 mAbs was detected after CASPR2 binding on transfected HEK cells. CASPR2 mAbs conjugated with the pH-sensitive dye pHrodo all showed strong signal after internalization into the acidic endosomal compartment after 24 h, with only HK 187–194 showing the same pattern already after 4 h (Fig. 2F). No signal was observed with the isotype control mAb mGO 53, nor in the untransfected condition after 24 h (Fig. 2F).

### 3.3 Human CASPR2 autoantibodies caused electrophysiological changes in vivo in rats

**Fig. 3:**
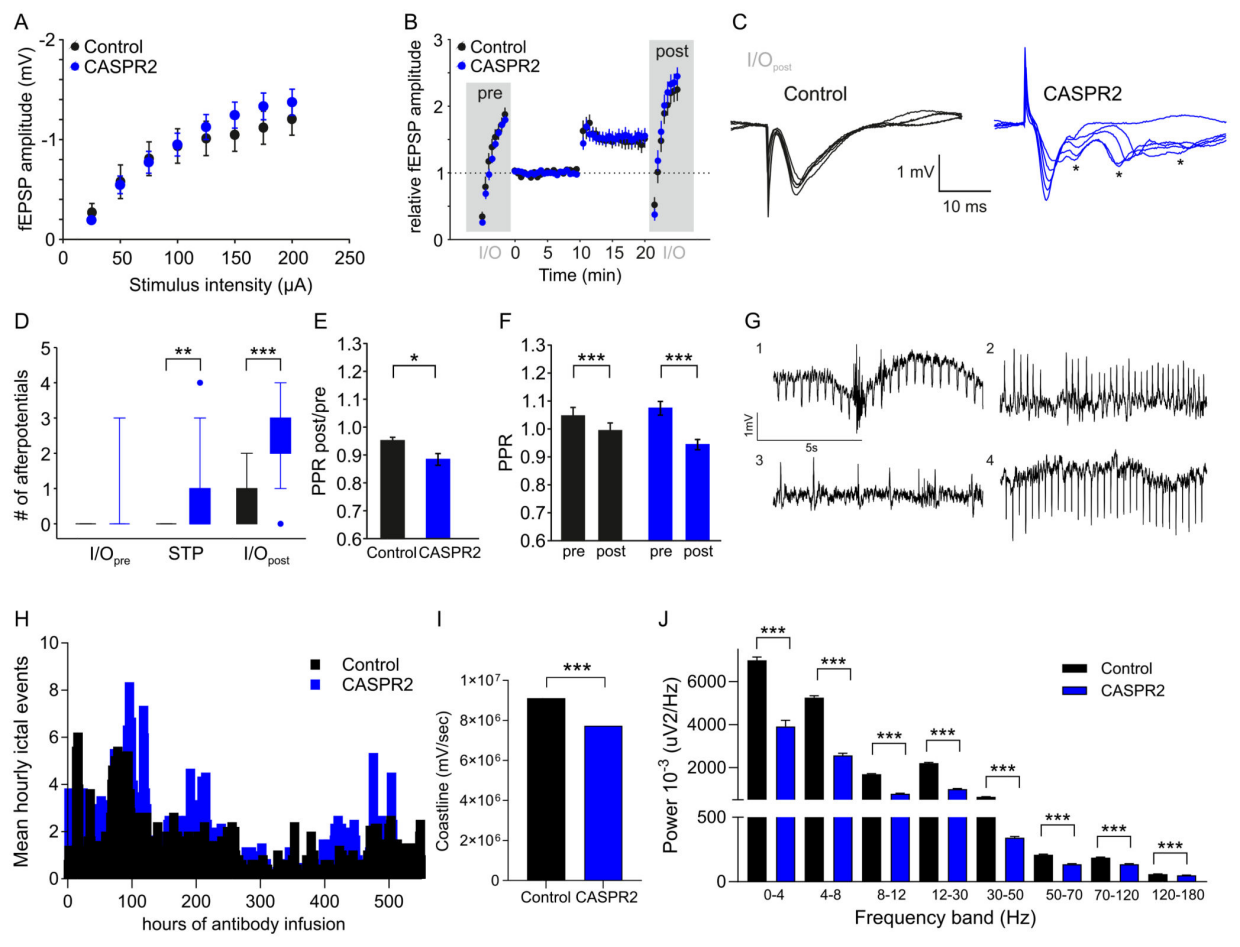
Electrophysiological effects of CASPR2 reactive monoclonal antibodies. fEPSP amplitude of ex vivo hippocampal slices measured after a range of stimuli intensities (25-200μA), for CASPR2 mab treated and control treated tissue (A). The relative fEPSP amplitude shown during, basal synaptic transmission input-out relationship (I/O_pre_) and potentiated synaptic transmission input-out relationship (I/O_post_) with STP induction at minute 10 (B). Representative fEPSP traces from a control and a CASPR2-mAb-injected animal, with epileptiform afterpotentials indicated with asterisks (C). Number of afterpotentials measured during I/O_pre_, STP induction and I/O_post_, with isignificant differences in the STP and I/O_post_ conditions between CASPR2 and control treated tissue (P<0,01 Mann-Whitney test) (D). The paired-pulse ratio (PPR) showed a significant decrease after STP induction in both groups (E), but this decrease was significantly more pronounced in the CASPR2 group (F). Representative EEG four of the CASPR2-mAb infused animals, 1) a hyper-excitability event seen on day 5 during the night (Supplemental video 1), 2,3,4) and when awake (G). Mean hourly 1-second ictal events seen over the total period of infusion, seen here for CASPR2 (n = 6) and control (n = 5) (H). Comparison of hourly measurements of coastline length between control (n = 5) and Caspr2 mAb (n = 6) infused rats over 21 day recording period (P≤0.0001; Mann-Whitney). Bars indicate mean ± SEM (I). Comparison of hourly EEG power averages in frequency bands shown between control (n = 5) and CASPR2 mAb (n = 6) infused rats over 21 day recording period (P≤0.0001; Mann-Whitney). Bars indicate mean ± SEM (J).

Based on its strong CASPR2 binding and inhibition of the interaction with contactin 2, we selected the CASPR2 mAb HK187-194 for further functional assays in rodent models. To assess the functional consequences of CASPR2 mAbs after *in vivo* stereotactic injection, we obtained extracellular recordings from hippocampal CA3 region. The input–output relationships did not differ between CASPR2 and PBS, neither before (I/O_pre_), nor after (I/O_post_) induction of short-term potentiation (STP; Fig. 3A-C). However, we observed epileptiform afterpotentials after STP induction and also during I/O_post_, significantly more so in slices from CASPR2 mAb-injected animals (p < 0.01, p < 0.001 Fig. 3A,D). In addition, while the paired-pulse ratio in both groups decreased significantly after STP induction (p < 0.05, Fig. 3E,F), the change in this ratio after STP was significantly greater in the CASPR2 mAb group (p < 0.001, Fig. 3F).

To understand whether the electrophysiological changes detected here at the molecular level could also lead to functional abnormalities, we further assessed EEG changes after 7 days of CASPR2 mAb infusion into lateral ventricles. Simultaneous continuous wireless depth EEG recording did not show spontaneous convulsive epileptic seizures but demonstrated evidence of hyperexcitability in all 6 animals (Fig. 3G, Supplemental video 1). Using automated ictal detection there was no significant difference in the number of ictal events (of 1 s duration) captured on EEG over the 21-day recording period between control and CASPR2 mAb-infused animals (Fig. 3H). Interestingly, a significant decrease in EEG coastline was observed in CASPR2 mAb-infused animals (p < 0.001), which was paired with reduced power in all EEG frequency wavebands (p < 0.001, Fig. 3I,J).

### 3.4 Intrathecally administered CASPR2 mAbs caused changes in functional MRI and social behavior in mice

**Fig. 4:**
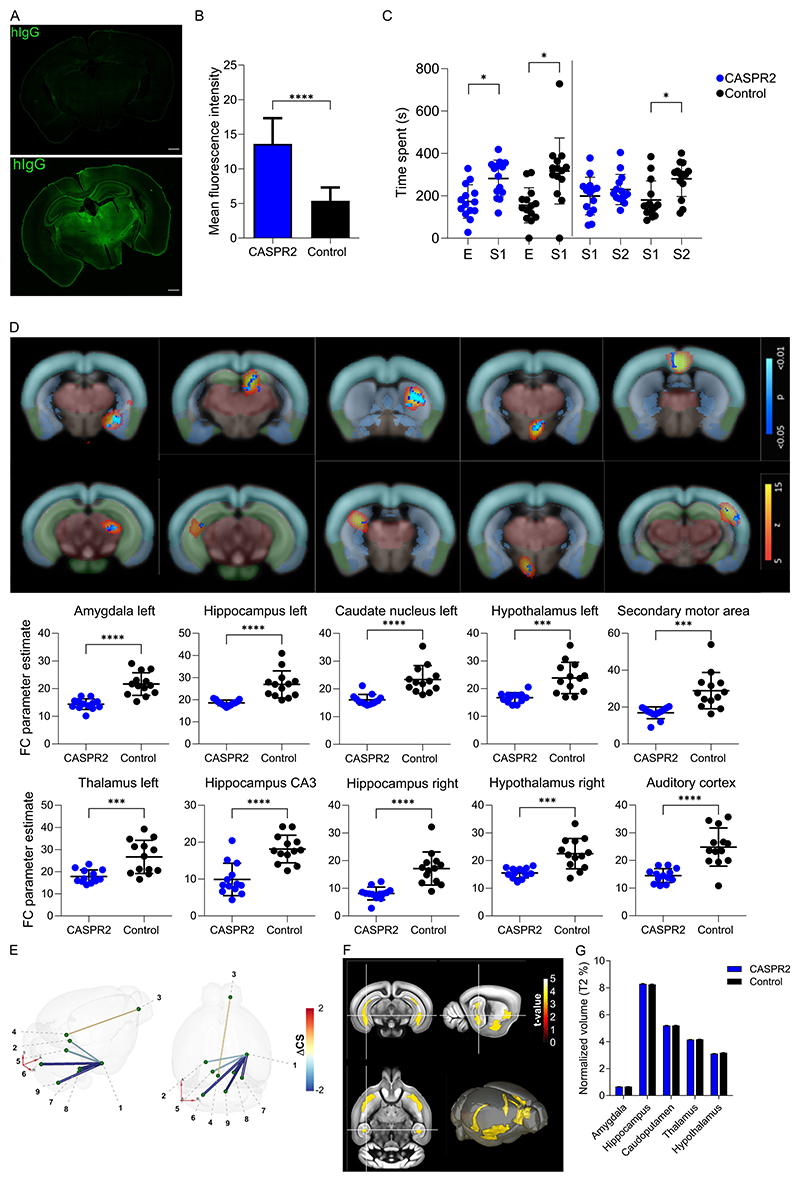
*In vivo* effects of infusion of CASPR2 reactive monoclonal antibody in mice. Representative tile scan image of coronal section of mouse infused with CASPR2 antibody or control after 14 days, with human IgG shown in green (A). Mean fluorescence intensity as measured in 10 areas, in 3 coronal slices of each animal on both the ipsi and contra lateral side to the injection site, for each animal (n = 6 for each group, p < 0.00001; *T* test) (B). Time spent at each stranger mouse ‘S1, S2′, with S1 serving as the familiar mouse in the novelty test, or empty cage ‘E’ after 10 days of infusion of either control or CASPR2 antibody in a three-chamber test paradigm (n = 14 for each group; p < 0.01; paired *t*-test) (C). Other behavioral paradigms tested can be found in supplemental Fig. 2. Functional connectivity group comparisons (CASPR2 > control, n = 14 each group) through assessed using dual regression as are shown superimposed on coronal sections of the Allen Mouse Brain Atlas (AMBA), with neo represented by coronal anatomical MRI scan image with cortex shown in light blue, hippocampus in green, thalamus area in red and mesencephalon in grey; ICA components in yellow–red; and significant group differences in blue-light blue. Extracted individual animal functional connectivity values are shown separately for both groups in scatter plots, in corresponding positions to results on AMBA sections (p < 0.001; *T* test; D). DTI analysis using the following predetermined clusters; 1) amygdala, 2) substantia nigra, 3) olfactory bulb, 4) periaqueductal gray, 5) trigeminal pontine nucleus, 6) trigeminal spinal nucleus, 7) trigeminal sensory nucleus, 8) superior olivary complex, 9) inferior olivary complex.Affected connections shown in a representative 3D model of a mouse brain, comparison represents CASPR2 vs Control (n = 14 each group), with the difference in CS shown in gradient (E). DTI cluster size analysis CASPR2 vs Control (n = 14 each group) with the difference shown in gradient within coronal, transversal, and sagittal cross section of a representative anatomical mouse scan (F). Brain area volume as measured by T2 anatomical scan for amygdala, hippocampus, caudatoputamen, thalamus and hypothalamus, after 14 days of infusion (n = 14 per group) (G).

For detailed characterization of clinically relevant effects in a mouse model of continuous intrathecal CASPR2 mAbs osmotic minipump infusion, mice received the human CASPR2 mAb HK187-194 over 14 days. Histological assessment confirmed strong binding of CASPR2 mAbs compared to control antibody in multiple brain areas including hippocampus, thalamus, and basal ganglia, correlating with the CASPR2 expression pattern (p < 0.0001, Fig. 4A-B).

Shortly before the end of mAb infusions, we evaluated behavioral effects of HK187-194 treatment using different paradigms (Suppl. Fig. 2A-D, Fig. 4C). CASPR2 mAb-treated mice showed deficits in social novelty in the three-chamber test. Both groups spent more time with another mouse (*S1*) compared to the empty cage (*E*), indicating unchanged sociability (p < 0.05). In contrast, the CASPR2 mAb group showed similar investigation of a novel intruder mouse (*S2*) compared to the familiar mouse (*S1*), indicating lack of interest in social novelty compared to the normal behavior of the control group (*S2* > *S1*) (Fig. 4C). Further behavioral assessments, did not result in a distinct overt phenotype of the CASPR2 mAbs-infused mice (Suppl. Fig. 2).

At experimental day 14, all animals underwent a comprehensive MRI protocol including anatomical and functional scans. The fMRI analyses demonstrated significant differences in the functional connectivity of multiple brain areas between CASPR2 and control antibody-infused mice (p < 0.0001, Fig. 4D). Mice receiving CASPR2 mAbs showed significantly lower functional connectivity in both the left and right hippocampus, caudate putamen, hypothalamus, and neocortical areas, such as the secondary motor area (Fig. 4D). Decreased connectivity was similarly observed between the amygdala and several connected brain areas in DTI analysis (Fig. 4E-F). Despite significant differences in fMRI and DTI analysis, no volume differences across brain areas in T2-weighted anatomical scans were found (Fig. 4G).

## 4 Discussion

In this study, we focused on pathogenicity of a panel of CSF-derived human CASPR2 mAbs from CASPR2 encephalitis patients. Intrathecal administration into rats or mice caused presynaptic electrophysiological abnormalities and afterpotentials *ex vivo* and resulted in a subtle clinical phenotype with alterations in EEG, fMRI and behavior *in vivo*.

### 4.1 Pathogenicity of anti-CASPR2 mAbs in vitro

A proposed pathogenic mechanism of CASPR2 autoantibodies is the disruption of CASPR2/contactin 2 interaction, leading to dislocation of the VGKC complex (Patterson et al., 2018). Indeed, the here characterized CASPR2 mAbs interfered with the binding between CASPR2 and contactin 2 in an affinity- and concentration-dependent manner. SPR measurements demonstrated the high affinity to the underlying autoantigen, which has not been described previously in the context of autoimmune encephalitis. Thereby, all CASPR2 mAbs targeted a restricted antigenic region, the CASPR2 discoidin domain, which has also been the most common target in previous studies using polyclonal CSF (Liang et al., 2019) or serum (Olsen et al., 2015). We cannot conclude yet how binding to the discoidin domain mediates the interruption of CASPR2/contactin 2, however, structural resolution of this protein complex will provide an answer in the future, similar to a cryoelectron microscopy study using GABA_A_ receptor (GABA_A_R) mAbs (Noviello et al., 2022).

Pathogenicity can further result from internalization of CASPR2 through antibody binding. Previous studies using polyclonal IgG have yielded mixed results regarding whether CASPR2 autoantibodies can induce internalization. Here we demonstrate that anti-CASPR2 mAbs were indeed internalized after CASPR2 binding, consistent with previous work on CASPR2 internalization in the hippocampus using serum-derived anti-CASPR2 antibodies (Joubert et al., 2022).

### 4.2 Clonal characterization of patient-derived mAbs

Beyond epitope analyses, characterization of disease-specific auto-antibodies at the clonal level allows for a number of further investigations, which have not been possible with polyclonal IgG preparations (Duong and Prüss, 2023; Noviello et al., 2022). For example, the here identified CASPR2 mAbs showed high numbers of SHM and evidence of clonal relationships, indicating antigen-driven affinity maturation. This aligns well with recent observations in one patient where CSF-derived CASPR2 autoantibody-producing B cells showed high numbers of SHM (Theorell et al., 2024). Similar patterns were observed in the CSF of patients with leucine-rich glioma inactivated 1 (LGI1) encephalitis, but not in N-methyl-D-aspartate receptor (NMDAR) encephalitis, where many mAbs showed few SHM or were even unmutated (Kornau et al., 2020; Kreye et al., 2016; Sun et al., 2020; Theorell et al., 2024). Furthermore, clonal relationships were recently found to enrich in CASPR2 reactive B cell populations, specifically in ASC dominated populations (Theorell et al., 2024). While IgG4 was the predominant subclass of the here described CASPR2 mAbs – in line with serum findings of CASPR2 encephalitis patients (Van Sonderen et al., 2016) – CSF-derived mAbs were mainly IgG2 in patients with LGI1 encephalitis and IgG1 in NMDAR and GABA_A_R encephalitis (Kornau et al., 2020; Kreye et al., 2021, 2016).

We further identified non-CASPR2 mAbs from CSF, comparable to previous studies where mAbs targeted yet unknown antigens on brain sections including neuronal surface antigens, neuropil epitopes, astroglia and vasculature (Kornau et al., 2020; Kreye et al., 2021, 2016; Theorell et al., 2024). Many of these mAbs originated from antibody-secreting cells, suggesting that they are also present in CSF and could contribute to brain dysfunction. Proof of concept for this hypothesis has been provided, for example, by antibodies against glucose-regulated protein 78 (GRP78) in addition to aquaporin 4 (AQP4) in patients with neuromyelitis optica spectrum disorder (NMOSD) (Shimizu et al., 2017) or antibodies against unconventional Myosin-X in NMDAR encephalitis (Li et al., 2023). Thus, ongoing work aims for the target identification of the underlying antigens to understand their contribution to autoantibody-mediated pathogenicity.

### 4.3 Anti-CASPR2 mAb were pathogenic in rodent models

As expected from human CASPR2 autoimmunity, the *in vivo* phenotype in rodent models after 14 days of intrathecal mAb infusion was relatively subtle compared to animal models using LGI1 or GABA_A_R mAbs, in which overt epileptic seizures occurred frequently(Kornau et al., 2020; Kreye et al., 2021). The subtlety likely relates to the relatively slow clinical progression of CASPR2 autoimmune encephalitis in humans compared to other encephalitides, and the absence of MRI, CSF and EEG changes in many cases(Benoit et al., 2023; Van Sonderen et al., 2016). Furthermore, the complexity of Morvan syndrome is hard to model, as previous studies failed to fully replicate the intricate phenotype(Baudin et al., 2023; Giannoccaro et al., 2019; Petit-Pedrol et al., 2018). The here reported CASPR2 mAb-associated deficits in social novelty correlated well with findings from a previous study of systemic (intraperitoneal) injection of human serum-derived CASPR2 antibodies together with increasing leakiness of the blood–brain barrier (Giannoccaro et al., 2019). In the same study, the observed phenotype had also been subtle. Similar to the unspecific symptoms early in the human disease, longer mAb exposure might have been needed for a stronger clinical phenotype to emerge(Benoit et al., 2023).

In line with the observed clinical phenotype in mice and rats, CASPR2 mAb-treated animals showed specific changes in *in vivo* EEG recordings, *ex vivo* electrophysiology and fMRI. The hyperexcitability observed *in vivo* and *in vitro* together with reduced power in all EEG frequency wavebands is different to other rodent models with epileptogenic activity where power is increased(Born et al., 2017; Kreye et al., 2021), similar to the well-known hyperexcitability in human patients with CASPR2 autoantibodies (Kirschstein and Köhling, 2023). EEG frequency band power is also a well-established metric in animal models and humans for various brain processing functions, such as learning, memory and sleep(Buzsáki et al., 2012). Specifically, disruption of sleep homeostasis can lead to the oscillatory changes as seen here, and sleep disruption is a cardinal feature of Morvan syndrome associated with CASPR2 autoantibodies(Binks et al., 2018; Irani et al., 2012; Vincent et al., 2018); detailed analysis of the rodent sleep video-EEG is the goal of future studies. Furthermore, the most dramatic reduction of EEG power was observed in the gamma frequency range. Perturbations of gamma oscillations have also been documented in genetic knockout models of CASPR2, linking immunogenic disruption to genetic disruption of CASPR2 function(Paterno et al., 2021).

Hippocampal slices from CASPR2 mAb-injected animals presented epileptiform afterpotentials *in vitro* implying that the hyperexcitability observed *in vivo* correlates to an epileptic network at the cellular level. Indeed, the present results confirm the increased excitability seen with patient-derived CSF in a model of acutely evoked epileptic discharges (Kirschstein et al., 2020). Since basal synaptic transmission and the STP magnitude were not altered in CASPR2 mAb-treated tissue, major changes to transmitter release and postsynaptic function are unlikely. However, our analyses revealed a significant difference in the paired-pulse ratio after STP induction, which clearly points to a presynaptic change in CASPR2 mAb-injected animals becoming unmasked only in the potentiated state. Since intrinsic excitability was not altered in CA1 pyramidal cells(Kirschstein et al., 2020) and CASPR2 is expressed on inhibitory axons(Pinatel et al., 2015) we conclude that CASPR2 antibodies in the present study impacted on interneuronal axons presynaptic to CA3 principal cells rather than on the A/C fiber presynaptic terminal. This, in turn, is consistent with a change in the extended network around CA3 pyramids, but not with a change in the A/C fibre-CA3 synapse itself.

Using fMRI, CASPR2 mAb-treated animals showed a marked decrease in functional connectivity compared to controls, for example in hippocampus and cortical areas. Human MRI findings similarly indicate altered functional connectivity in patients with mutations in CASPR2 (Dennis et al., 2011; Scott-Van Zeeland et al., 2010). Although connectivity changes are highly plausible given decreased neurite lengths and reduced numbers of dendritic spines in CASPR2 knockout animals (Anderson et al., 2012), fMRI studies in models of autoimmune encephalitides have just emerged (Kuchling et al., 2022), and the detailed underlying mechanisms that link neuronal dysfunction to fMRI paradigms are yet to be explored. The successful application of minimally invasive (EEG) or non-invasive (fMRI) techniques suggests, however, that further validation can lead to improved objective and quantifiable animal models, in particular if the expected phenotype consists of subtle neuropsychiatric or memory abnormalities, detection of which is naturally limited by the lower complexity of the rodent brain.

### 4.4 Limitations

The present study demonstrates pathogenicity of CASPR2 mAbs, however, the small number of patients who were all elderly males, and the small number of recombinant mAbs limits generalizability of the findings. Cloning of further mAbs from patients with different clinical subtypes will be needed to determine the frequency of autoantibodies targeting epitopes outside the discoidin domain, the ability to block interaction with contactin 2, the contribution to clinical variability, symptom severity or treatment responsiveness. This study has focused on Fab-mediated pathogenicity, showing effects based on the binding of antibodies to CASPR2, however, other antibody mediated pathogenic mechanisms, such as complement activation and Fc receptor interaction, remain to be studied in greater detail (Chalayer et al., 2022; Coss et al., 2023).In addition, more detailed analyses of sleep behavior in treated mice seem promising, however, we did not correlate rodent EEG with simultaneous EMG, thus precluding accurate assessment of sleep stages.

### 4.5 Conclusion and perspective

Taken together, we provide evidence *in vitro* and *in vivo* that patientderived CASPR2 mAbs are directly pathogenic, can bind to the discoidin domain of CASPR2 with affinities in the pico- to nanomolar range, interfere with CASPR2/contactin 2 binding, and lead to robust changes in rodent models of CASPR2 encephalitis including hyperexcitability and widespread reduced functional connectivity. As CASPR2 mAbs are sufficient for pathogenic effects at the molecular level, abnormal CASPR2 function may also occur in autoantibody-positive patients without encephalitis, suggesting the potential contribution of low-level CASPR2 autoantibodies to further neuropsychiatric symptoms. Results from this study may have implications for the development of novel autoantibody-selective immunotherapies in the future, such as CAAR T cells and strongly support current treatment strategies including B cell depletion in CASPR2 encephalitis (Reincke et al., 2023).

## Supplementary Material

Supplementary data

## Figures and Tables

**Table 1:** Sequence information of isolated antibodies.

Antibody	Cell type	Ig subtype	HC V	HC J	KC/LC V	KC/LC J	SHM	Clonality	CASPR2 reactive
HK 187–113	ASC	IgG4	3–11	1	1–33	4*01	43	Expanded	+
HL 187–114	ASC	IgG1	3–21	6	1–51	3*02	4	–	−
HK 187–120	ASC	IgA1	4–34	6	1–17	1*01	2	Expanded	−
HK187–127	ASC	IgM	3–33	4	1–27	2*02	2	–	−
HK 187–147	ASC	IgA1	4–34	6	1–17	1*01	2	Expanded	−
HK 187–155	ASC	IgG4	4–39	6	3–15	5*01	10	–	−
HK 187–160	ASC	IgM	3–07	6	1–39	2*01	40	–	−
HL 187–180	ASC	IgG4	3–23	4	1–47	2*01	19	–	+
HL 187–188	ASC	IgG2	5–51	4	1–47	3*02	27	–	+
HK 187–194	ASC	IgG4	3–11	1	1–33	4*01	43	Expanded	+
HK 219–142	ASC	IgG4	4–59	6	3–20	1*01	35	–	+

Germline gene segments for V, D and J are listed according to highest homology for both the Heavy chain (HC) and kappa/lambda chain (KC/LC). The number of somatic hyper mutations (SHM) shown represents the total mutations present in both the heavy and light Ig chain of each antibody. Expanded clonality indicates presence of another antibody with identical V(D)J gene segments for both heavy and light, as well as identical mutation counts and patterns. CASPR2 reactivity shows reactivity against human CASPR2 as performed in a cell based assay.

## Data Availability

Data will be made available on request.
