# Supplementary material for: Human cerebrospinal fluid monoclonal CASPR2 autoantibodies induce changes in electrophysiology, functional MRI, and behavior in rodent models
Source: Brain Behav Immun. Author manuscript; Available in PMC 2025 Sep 4. (PMC7618075; doi:10.1016/j.bbi.2024.08.027)
Supplement: Supplementary data [file EMS208257-supplement-Supplementary_data.zip › 1-s2.0-S088915912400552X-mmc1.docx]

**Supplementary Table 1**

*Patient characteristics.*

| Patient ID | Age, Gender | Clinical presentation | Disease phase | Immunotherapy at time of CSF puncture | Titer Serum | Titer CSF |  |  |  |  |  |  |  |  |  |  |  |  |  |  |  |
| --- | --- | --- | --- | --- | --- | --- | --- | --- | --- | --- | --- | --- | --- | --- | --- | --- | --- | --- | --- | --- | --- |
| 187 | 66, male | Morvan-Syndrome | Acute encephalitis | No treatment | 1:1000 | 1:1000 |  |  |  |  |  |  |  |  |  |  |  |  |  |  |  |
|  |  | Myokymia |  |  |  |  |  |  |  |  |  |  |  |  |  |  |  |  |  |  |  |
|  |  | Cognitive disturbance |  |  |  |  |  |  |  |  |  |  |  |  |  |  |  |  |  |  |  |
| 219 | 60, male | Ataxia | Remission phase | Plasmapheresis | >1:10.000 | 1:100 |  |  |  |  |  |  |  |  |  |  |  |  |  |  |  |
|  |  | Myoklonus |  | IVIg |  |  |  |  |  |  |  |  |  |  |  |  |  |  |  |  |  |
|  |  | Memory loss |  | Prednisolon |  |  |  |  |  |  |  |  |  |  |  |  |  |  |  |  |  |
|  |  | Change of character |  | Rituximab |  |  |  |  |  |  |  |  |  |  |  |  |  |  |  |  |  |
|  |  |  |  | Bortezomib |  |  |  |  |  |  |  |  |  |  |  |  |  |  |  |  |  |
|  |  |  |  | Daratumumab |  |  |  |  |  |  |  |  |  |  |  |  |  |  |  |  |  |

*Patient characteristics for two patients diagnosed with anti-CASPR2 autoimmune encephalitis. Clinical presentation is shown at the time of admittance. Immune therapy is indicated for the time of lumbar puncture. Both serum and CSF titer are shown for the timepoint of lumbar puncture. Status of encephalitis and treatment shown at the time of lumbar puncture from which the CSFC’s are isolated.*
